# Formal Childcare Use and Mothers’ Fertility Intentions and Behaviours: Evidence in Italy by Migration Background

**DOI:** 10.1007/s10680-025-09746-6

**Published:** 2025-11-10

**Authors:** Eleonora Miaci, Eleonora Mussino, Eleonora Trappolini, Giammarco Alderotti, Cristina Giudici

**Affiliations:** 1 Department of Statistical Science, Sapienza University of Rome, Rome, Italy; 2 Stockholm University Demography Unit (SUDA), Stockholm, Sweden; 3 Department of Statistics, Computer Science, Applications, University of Florence, Florence, Italy; 4 Department of Sociology, Umeå University, Umeå, Sweden; 5 Department of Methods and Models for Economics, Territory and Finance, Sapienza University of Rome, Rome, Lazio Italy

**Keywords:** Migrants’ fertility, Fertility intentions, Fertility behaviours, Formal childcare services, Unmet need for childcare, Italy

## Abstract

Numerous studies have explored the influences of family policies, such as formal childcare use, and migration on fertility, with mixed findings. However, limited research has examined how formal childcare use (or the lack thereof) affects both fertility intentions and behaviours among native and migrant women. This study uses Italy as a case study, where the *familistic* welfare system creates challenges in work–family reconciliation and female workforce participation, particularly for migrant women facing precarious jobs and higher fertility. This results in employment disparities between migrant and native women, especially among mothers. Using the 2012 Birth Sample Survey from the Italian National Institute of Statistics, we address two research questions: (i) Does formal childcare use for one child positively influence mothers' fertility intentions and behaviours to have another? (ii) Does this effect vary according to migration background? We apply multinomial logistic regression models to analyse the relationship between mothers' fertility intentions and behaviours and childcare use by migration background, migratory generation, and partner's nationality. Our results show that mothers using formal childcare—either stable or occasional—are more likely to have positive fertility behaviours than those with unmet needs, with differences by migration background. Among mothers using formal care, natives show higher positive short-term fertility intentions than migrants, while natives with unmet childcare needs are less likely to have another child than migrants. While formal childcare has limited effect on fertility, unmet childcare needs emerge as a crucial factor, highlighting the need for policies addressing broader socio-economic and cultural factors shaping fertility decisions.

## Introduction

Understanding how families organise childcare is crucial due to its strong association with women's participation in the workforce (Del Boca & Vuri, 2007) and its potential impact on forthcoming fertility choices and behaviours (Rindfuss et al., 2010). Research shows a positive relationship between childcare use and fertility, indicating that formal childcare helps balance employment and family life (Andersson et al., 2004; Del Boca, 2002; Dimai, 2023; Fiori, 2011; Mathews & Sear, 2013). However, some of these studies have focused on fertility behaviours, while others have concentrated on fertility intentions, leading to different conclusions.

Other research has examined the link between limited access to paid or formal care services and negative outcomes later in life, such as cognitive disorders or physical disabilities (Broese van Groenou et al., 2006; Vlachantoni et al., 2015).

To our knowledge, no studies have investigated the impact of both the use of formal childcare and unmet childcare needs simultaneously on fertility intentions and behaviours. To bridge this divide, the first study's aim is to adopt a comprehensive approach, using a dependent variable that integrates both reproductive intentions and actual behaviours of the respondents. We will examine the association between the childcare use (for one child) or the unmet need for childcare and mothers' fertility intentions, as well as the short-term realisations within 2 years after a previous child's birth.

Most research in this area primarily focuses on the general population and commonly views migrants as caregivers rather than potential users of childcare services (Williams & Gavanas, 2016). As migrants settle in their new host country, reunite with family members, or form new family units, childcare becomes essential for balancing employment and parental responsibilities (Trappolini et al., 2023). Recently, scholars (e.g. Mussino & Ortensi, 2023) have highlighted a gap in our understanding regarding the extent to which family policies are inclusive or exclusive towards international migrants and the subsequent effects on their fertility decisions. This issue is particularly relevant in Italy for several reasons. First, Italy's welfare system is characterised by a *familistic* model of care based on solidarity between generations, resulting in a lack of supportive measures and public services that aid in balancing work and parental responsibilities and promoting women's integration into the workforce (Esping-Andersen, 1990, 2016; Santero & Naldini, 2020). Second, Italy also faces low fertility rates, a trend observed even among much of the migrant population (ISTAT, 2022a). Migrant groups, characterised by distinct socio-demographic profiles compared to the native population, often struggle more in accessing adequate childcare services (Mussino & Ortensi, 2023). Third, the male breadwinner model, historically embedded in the Italian care system, has resulted in childcare responsibilities falling predominantly on women (Mencarini & Solera, 2011; Naldini & Saraceno, 2011). Within this framework, migrant status further restricts access to childcare options, limiting workforce participation of migrant women, particularly mothers (Barbiano di Belgiojoso et al., 2023). Thus, the second aim of this study is to address a notable gap in the existing literature by examining whether the relationship between formal childcare use or unmet need for it and fertility varies by migrant background. Additionally, the study's third aim is to explore the complexity and heterogeneity of migrant populations. By acknowledging the limitations of a binary distinction between native-born and foreign-born mothers, we consider variables that account for migratory generations and the combination of parents' migration background.

We used data from the third and latest edition of the Sample Survey on Births (“*Indagine campionaria sulle nascite e le madri*”), conducted by the Italian National Institute of Statistics (ISTAT) in 2012. Mothers were interviewed about their use of and unmet need for formal childcare. Using multinomial logistic regression models, we analyse the association between mothers' fertility intentions and behaviours and their use of formal childcare services.

The present study is the first to adopt a comprehensive approach that investigates both fertility intentions and actual behaviours, while also assessing the impact of unmet childcare needs on native and migrant mothers in Italy.

## Conceptual Framework

### Family Policies and Their Impact on Fertility Intentions and Behaviours

Scholars have mainly focused on the effects of family policies on gender equality (Adema, 2013; Haas, 2003; Niemistö, 2011), women's labour market integration (Del Boca 2002; Del Boca & Vuri, 2007; Ellingsæter, 2009), and positive outcomes for children's development, academic performance, and social integration (Bergamante & Solera, 2019; Blanden et al., 2022; Karhula et al., 2017; Saraceno, 2011). In addition, several studies have explored how family policies, including childcare services, influence fertility intentions and behaviours separately (Dimai, 2023; Fiori, 2011; Luci-Greulich & Thévenon, 2013). The key finding from this body of research is that measures such as financial transfers, parental leave, and childcare services can positively influence both fertility intentions and behaviours by reducing the financial burden of childrearing and helping balance work and family life. However, different measures may have different effects on fertility, and the magnitude of the positive effect of each policy lever remains moderate (see, e.g. Guetto et al., 2025). For instance, Bergsvik et al. (2021), in a review of experimental studies from Europe, the USA, Canada, and Australia, found that expanding childcare availability tends to have a more significant and long-lasting impact on fertility than financial transfers. When it comes to childcare services, other studies showed that increased childcare availability boosts the likelihood of having children (e.g. Del Boca, 2002). However, findings are mixed: Some studies report no significant effect on the transition to motherhood—for example in Western Germany (Hank & Kreyenfeld, 2003)—while others indicate limited but positive short-term effects (see, e.g. Scherer et al., 2023 for Italy).

The mixed findings on the relationship between childcare availability and fertility suggest that family policies may operate differently depending on broader structural conditions and individual-level circumstances. This underscores the need to distinguish between macro- and micro-level effects when assessing how family policies shape fertility outcomes. At the macro-level, several studies demonstrated that countries with robust family policies and affordable childcare services tend to have higher fertility rates (Di Giulio & Pinnelli, 2007; Neyer, 2006). At the micro-level, findings are more varied due to differences in research design, sample selection, and operational definitions. For example, some studies focussed on respondents with only one child (Fiori, 2011, exploring fertility intentions; Mathews & Sear, 2013, exploring behaviours), while others included individuals with higher-order children (Del Boca, 2002; Andersson et al., 2004, exploring fertility behaviours; Cheng & Hsu, 2020, exploring fertility intentions). Many studies limited their sample to women in relationships (Del Boca, 2002; Andersson et al., 2004, exploring fertility behaviours; Fiori, 2011; Cheng & Hsu, 2020, exploring fertility intentions), though some have included a broader range of respondents (Mathews & Sear, 2013, exploring fertility behaviour). Other studies have stratified their samples by employment status (Del Boca, 2002, exploring behaviour; Fiori, 2011, exploring fertility intentions) or respondents' socio-economic characteristics (Cheng & Hsu, 2020, exploring fertility intentions).

Another key source of divergence among studies is the type of childcare regimes considered (Andersson et al., 2004). In the US and Anglo-Saxon countries, the emphasis has been on childcare cost and quality (Blau, 2001; Ermisch, 1989), while in Europe, where services are more often publicly funded, the focus has been on the availability of childcare (Baizan et al., 2016; Del Boca, 2002; Wood & Neels, 2019). There is considerable heterogeneity between countries: Nordic countries provide universal childcare, whereas southern and eastern European countries tend to place a greater burden on families (Saraceno, 2011). Additionally, many studies have explored the determinants and consequences of unmet childcare needs (e.g. Brilli et al., 2016; Mussino & Ortensi, 2023; Naldini & Santero, 2019; Naldini & Saraceno, 2022), investigating how these vary by geographical location (Levesque et al., 2013), gender norms and roles (Miller & Bairoliya, 2020; Sevilla-Sanz et al., 2010), and the relationship between household socio-economic status, education, and unmet childcare needs (Craig, 2006; Broese van Groenou et al., 2006; Vaghul, 2019; Rodrigues et al., 2018).

### Fertility and Childcare in Italy: Differences by Migrant Background

Our study does not aim to reconcile the heterogeneity of results through comparative analysis, but focuses on the Italian context, providing significant insights into the effects of childcare use on fertility short-term intentions and behaviours.

Migrant women have contributed to mitigating the decline in birth rates due to their significant presence at fertile ages and younger average age at childbirth, with fertility rates close to replacement levels (1.86 children per woman in 2022b). In 2022, approximately 13.5% of births in Italy were to foreign-born parents and 20.9% to couples with at least one foreign-born partner.

Mixed couples, with at least one foreign-born partner, exhibit distinct fertility patterns compared to endogamous couples in Italy. Traditionally, in Italy, mixed couples tend to have lower and later fertility, higher abortion rates, and more previous marriages and children (Maffioli et al., 2012). However, fertility trends among migrants and natives are increasingly converging, possibly due to systemic barriers affecting reproductive choices. These barriers are particularly challenging for migrant populations, especially women, who face precarious employment and difficulties in balancing work and family life (Bonizzoni, 2015; Barbiano di Belgiojoso et al., 2023).

Moreover, migrant women may face an even more restrictive barrier known as the “maternal wall”, a set of stereotypes and discrimination encountered by working or job-seeking mothers. This barrier is particularly pronounced in certain migrant communities where cultural norms or limited access to support services make employment among mothers less common compared to Italians (Coccia et al., 2023). Data on employment rates reinforce these observations: While migrant women generally have higher employment rates than Italian women (59.3 vs. 56.1%), migrant mothers have lower employment rates than their Italian counterparts (43.6 vs. 59.8%) (ISTAT, 2019a). Additionally, migrant families have a median income approximately 6000 euros lower than families native Italians (ISTAT, 2023).

This socio-economic landscape leads to differences in childcare service use between natives and migrants. Recent research highlights that migrants face greater risks of lacking suitable childcare options (Mussino & Ortensi, 2023; Trappolini et al., 2023, 2024), often due to structural barriers, such as language difficulties and administrative complexities (Frazer et al., 2020; Karoly & Gonzales, 2011). While some migrants express a preference for formal childcare—varying significantly based on country of origin and the nationality of their co-parent (Mussino & Ortensi, 2023)—others rely on it out of necessity, given their limited access to informal options after leaving behind close family networks (Bojarczuk & Mühlau, 2018).

A key structural factor shaping these childcare choices is Italy's immigration reunification laws, which prioritise the nuclear family unit and often limit the availability of extended family members. This constraint frequently pushes migrant couples towards formal childcare arrangements, even when informal care might otherwise be preferred (Bergamante & Solera, 2019; Chinosi, 2002). However, when grandparents or other relatives are present and in good health, they often become the primary caregivers, highlighting how childcare decisions reflect a complex interplay between preference and necessity (Del Boca et al., 2005; Trappolini et al., 2021, 2023).

Among migrants, 1st-generation individuals face significant disadvantages both in accessing daycare facilities and due to having smaller support networks (Mussino & Ortensi, 2023). In the context of mixed couples, where one partner is native and the other is a migrant, childcare service utilisation follows a unique turn. According to Mussino and Ortensi (2023), these couples find themselves in an intermediate position. They are more likely than couples with two native partners to enrol their children in daycare, yet they face challenges similar to those faced by migrant couples, such as limited support networks. However, the presence of a native partner can facilitate navigation of the service system and may provide access to broader family-based support—such as grandparents—than is typically available to couples where both partners are migrants.

### Determinants of Fertility Intentions and Behaviours

The literature has identified numerous determinant factors influencing fertility intentions and behaviours. While these are not the primary focus of our paper, it is important to acknowledge and include them in our analysis as control variables.

We control for age, a key factor in fertility research, as both intentions and outcomes vary nonlinearly with age—being typically lower at younger and older ages, and peaking in intermediate reproductive years (Liefbroer, 2009). Parity also plays a significant role in shaping fertility. Higher parities correlate with reduced likelihoods of positive fertility intentions and behaviours (Begall & Mills, 2011; Billari et al., 2009; Brzozowska et al., 2022), particularly in a country like Italy, where the fertility rate is significantly below the replacement rate (1.24 in 2022, ISTAT, 2022b). Partnership status is another key determinant. Compared to those in partnership, singles are much less likely to have positive short-term fertility behaviours and intentions (Billari et al., 2009; Philipov et al., 2006).

Educational levels have been consistently associated with fertility, yet this relationship shows considerable variation across different European countries, within various demographic groups, and particularly among individuals with migrant background (Kulu et al., 2017; Carlsson, 2019; Mussino et al., 2021). In Italy, education increasingly affects fertility behaviours among younger cohorts, contributing to delayed first childbirth or childlessness among highly educated women, yet positively impacting second childbearing among those who become mothers (Impicciatore & Zuanna, 2017).

Economic disadvantages, in terms of low household income and wealth, discourage the decision to have a first child due to insufficient means to manage potential future adversities (Modena et al., 2014).

Employment instability generally has a negative effect on fertility, particularly in southern European countries with less generous social protection for families and the unemployed (Alderotti et al., 2021 for a review). Unemployment's effect varies by the gender of the unemployed partner (Adsera, 2011), and the presence or absence of social networks individuals can rely on (Keim, 2011; Rutigliano, 2020).

We also control for macro-area of residence to account for persistent regional heterogeneity in socio-economic conditions and demographic dynamics in Italy (Reynaud et al., 2020). Fertility has long varied across macro-areas, with earlier and sharper declines in the North and Centre. More recently, levels in these areas have overtaken those in the South, partly due to higher female employment and more dual-earner households (Alderotti, 2022; ISTAT, 2024). Regional differences also emerge in migrants’ fertility, which is lower in the South due to a smaller and older migrant population (ISTAT, 2022b; Miaci, 2025).

The literature is increasingly identifying differences in fertility behaviours and intentions between natives and migrants, even after accounting for demographic variables and socio-economic characteristics (Milewski, 2010; Kulu et al., 2017; Andersson et al., 2017; Carlsson, 2019; Alderotti et al., 2023). International literature identifies five main hypotheses, not mutually exclusive, to explain differences in migrant fertility patterns: socialisation, adaptation, selection, interrelation, and disruption (see Kulu et al., 2019 for a review). While, in this study, we do not aim to test all these hypotheses, we apply the socialisation and adaptation frameworks to explore how childcare use influences fertility intentions and behaviours, considering the migrant generation and the combination of parents' migrant background.

The socialisation hypothesis suggests that the migrants' fertility behaviours reflect the norm prevalent in their childhood environment. As a result, migrants are expected to show fertility levels similar to the residents in their country of origin (e.g. Garssen & Nicolaas, 2008). In contrast, the adaptation hypothesis posits that migrants' fertility behaviours tend to align with the destination country over time (e.g. Andersson et al., 2004).

On an operational level, to test these theories in different contexts, researchers commonly identify and analyse specific determinants. For instance, they often use the country of birth (or citizenship) as a proxy for the influences of the context of origin (Carling, 2005; Andersson & Scott, 2007; Mussino et al., 2015). Another critical factor to study the effect of exposure at destination is age at arrival, which is directly linked to the migrant generation. Arriving at a younger age is associated with weaker adherence to origin country norms due to less exposure to those cultural patterns, compared to individuals who migrate during adulthood and are likely to retain these norms (Toulemon, 2004; Mussino & Strozza, 2012; Mussino & Ortensi, 2023).

Studies exploring the fertility of mixed unions are predominantly conducted in the U.S. (Choi & Goldberg, 2018; Fu, 2008; Qian & Lichter, 2021), but sparse in Europe (Elwert, 2023; García-Pereiro et al., 2023; Mussino & Strozza, 2012). In the US, studies indicate that fertility rates in mixed unions are generally lower than those in couples with partners of the same migrant or ethnic background. This is often attributed to lower societal acceptance of interracial marriages, leading to less social support and, consequently, higher financial and emotional costs of childbearing (Fu, 2008; Qian & Lichter, 2021).

In Sweden, Elwert (2023) examined third-birth risks according to the sex composition of previous children and found that exogamous couples differ significantly from endogamous couples. Unions between Swedish women and immigrant men generally have higher third-birth risks, while unions between Swedish men and immigrant women show lower third-birth risks than endogamous Swedish couples.

Research in Italy suggests that having an Italian partner may facilitate the fertility adaptation of migrants to local fertility behaviours (Mussino & Strozza, 2012) and expanding their social network. Regarding fertility intentions, García-Pereiro et al. (2023), while testing the socialisation or adaptation hypotheses—evaluating whether foreign-born women partnered to Italian men will resemble, respectively, those of foreign-born women partnered to foreign-born men or, instead, those of Italian women partnered to Italian men—found support for the adaptation hypothesis, albeit with differences depending on migrants' origin.

## Research Hypotheses

Building upon the aforementioned literature, we formulate the following hypotheses:

### H1

We expect that the use of formal childcare services positively influences fertility intentions and behaviours of mothers in Italy, whereas unmet childcare needs deter both the intention to have another child and actual childbearing.

### H2

Furthermore, we hypothesise that formal childcare use has a stronger influence on short-term fertility intentions and realisation than on long-term fertility intentions, as short-term decisions should be more dependent on accessible childcare.

### H3

The influence of formal childcare use and unmet childcare needs on fertility intentions and behaviours differs between migrant and native mothers due to varying socio-economic, cultural, and institutional contexts. Specifically, we expect:

- H3a. Formal childcare use to have a stronger positive influence on fertility outcomes among natives than among migrants, as natives tend to rely more on formal childcare services.
- H3b. Unmet childcare needs to have a more negative influence on migrant mothers' fertility intentions and behaviours than on those of native mothers, due to the additional challenges migrants face.

### H4

Additionally, we expect 1st-generation migrant mothers, who tend to adhere more to traditional gender roles and exhibit less planned fertility, to be less responsive to formal childcare services than 1.5-generation migrant mothers, who are more adapted to the host country's childcare norms.

### H5

Finally, we expect mixed couples (one native and one migrant partner) to experience a more positive impact from formal childcare services on their fertility outcomes than migrant–migrant couples, yet benefit less than native–native couples, reflecting partial advantages in navigating childcare systems.

## Data and Research Sample

We used data from the 2012 Birth Sample Survey—“*Indagine campionaria sulle nascite e le madri*”—carried out by ISTAT.^1^ The interviewees were mothers of children born between the second half of 2009 and the first half of 2010. The interviews took place in 2012, about 18–21 months after giving birth, and mothers were asked about their intentions regarding having another child and their use of formal childcare services.

The survey employed two different data collection methods: computer-assisted telephone interviews (CATI) for the “Italian-born” group (i.e. individuals with at least one parent holding Italian-citizenship) and face-to-face interviews using a paper questionnaire (PAPI) for the “foreign-born” group (i.e. individuals whose parents both held foreign-citizenship). It should be noted that although this classification is based on parental citizenship, in our analysis, we defined migrants on the basis of their place of birth. We do not have detailed information on migrants' countries of birth; we only know whether individuals were born abroad. The survey records only their last country of residence before moving to Italy.

For study purposes, we excluded respondents without a partner/spouse, in line with prior research (Billari et al., 2009; Fiori, 2011; Rinesi et al., 2011), reducing our sample size from 19,256 to 18,321 mothers who gave birth to a child between 2010 and 2011.

Furthermore, we decided to focus on those who send their children to daycare and those who report an unmet need for childcare. For this reason, we excluded from the analysis mothers who reported being non-users of formal childcare by choice, as this group is likely influenced by different factors.

The final sample consisted of 8661 respondents. Among them, 45% of mothers were interviewed for the birth of their first child, while 55% were interviewed for higher-order births; mothers of twins were counted only once. Sample weights were applied.

It is important to highlight that this national survey includes data exclusively on individuals who are legally residing in Italy.

## Measurement

### Dependent Variable

Our dependent variable combines intentions and actual short-term fertility behaviours, assessed through a survey conducted 18–21 months postpartum. Approximately 12% of mothers were pregnant or had given birth again at the time of the interview, leading us to include them in our sample, diverging from previous research strategies (Carlsson, 2019; Fiori, 2011). We combined this information with the two questions related to intentions. The first question is “*Do you intend to have more children in the future?*”—and mothers who answered “Probably or Definitely not” were assigned to level 0 “Negative intentions”. Mothers answering “Yes” were further asked “*Within 3 years?*”—and if they responded “Probably or Definitely not”, they were classified as having “Positive life-time fertility intentions”. Respondents who answered “Probably or Definitely yes” were categorised as having “Positive short-time fertility intentions”.

Therefore, our final dependent variable has four categories: 0 for “Negative fertility intentions”, 1 for “Positive lifetime fertility intentions”, 2 for “Positive short-time fertility intentions”, and 3 for those “Pregnant or who gave birth after the initial child discussed in the interview”. This approach allows a nuanced analysis beyond a simple binary framework, incorporating the immediacy and specificity of intentions within a 3-year timeframe. Details on the distribution and categorisation of the dependent variable are found in Table 1, in the next section, Table 2 and Fig. 6 in the Appendix.

**Table 1:** Distribution of the main explanatory variables across the categories of the dependent variable

% weighted	Negative Intentions	Positive lifetime intentions	Positive short-time intentions	Positive short-time realisations
*Childcare use*				
Use of formal childcare	49.6%	3.7%	33.6%	13.1%
Suboptimal arrangement	49.6%	3.7%	35.7%	11.0%
No one to ask	53.2%	5.2%	25.3%	16.3%
*Migrant background*				
Foreign-born	42.9%	4.5%	31.9%	20.7%
Italian-born	53.1%	3.7%	32.6%	10.6%
*Migrant generations*				
1st-generation	44.1%	3.7%	31.9%	20.3%
1.5-generation	38.5%	7.3%	31.8%	22.5%
Natives	53.1%	3.7%	32.6%	10.6%
*Parents' migrant background*				
Both partners natives	53.4%	3.6%	32.4%	10.6%
Mother native/Father migrant	44.6%	5.3%	38.9%	11.2%
Mother migrant/Father native	49.8%	3.8%	34.6%	11.8%
Both foreign-born	40.7%	4.7%	31.1%	23.5%
*Parity at interview*				
1	22.5%	5.9%	61.8%	9.9%
2	70.6%	2.7%	12.5%	14.3%
3 or more	79.0%	1.1%	5.7%	14.2%
Share of observations	51.3%	3.8%	32.5%	12.4%
*N* (Number of observations)	9263	712	6071	2275

Note: Percentages are weighted and should be read by row

Source: Authors' elaboration on Birth Sample Survey/Indagine campionaria sulle nascite e le madri (2012)

### Main Explanatory Variables

We used two main explanatory variables:

1. mother's use of formal childcare (1 “Use of formal childcare”, 2 “Suboptimal arrangement”, and 3 “No one to ask”)
2. migration background

**Fig. 1:**
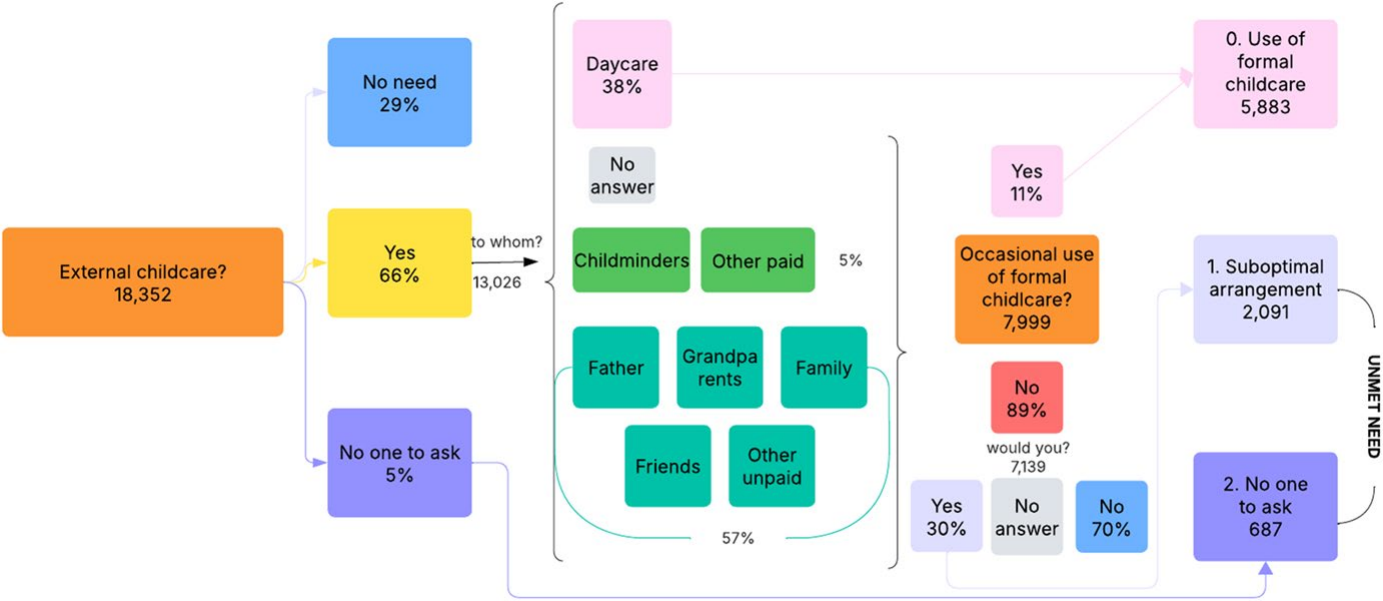
Survey question flow for the variable “mother's use of formal childcare”. *Note*: Percentages are weighted. *Source*: Authors' elaboration on Birth Sample Survey/Indagine campionaria sulle nascite e le madri (2012)

The first variable specifically addresses the mother's use of formal childcare services for the child born within the observation period. The distribution of the dependent and independent variables across the categories of the childcare use variable is in the Appendix (Table 5). Figure 1 illustrates how the variable was operationalised from several questions asked in the survey.

This information was derived from a single question: “*Do you rely on childcare provided by services or other persons*?” with different possible answers including “Yes”, “No, I have no need for childcare”,^2^ and “No, I have no one to ask”. Respondents selecting the last option were classified under the “*No one to ask*” category.

For mothers who answered “Yes”, the survey further asked the specific childcare they use: “*To whom is the child primarily entrusted?*” The available options include different types of childcare services; however, mothers were allowed to provide only one choice.

If the mother chose “Daycare” (formal childcare), she fell into the “*Use of formal childcare*” category.

For mothers who entrusted their child to other forms of childcare, such as childminders, partners, friends, neighbours, or grandparents, an additional question was asked: “*Do you occasionally use formal childcare services?*” A positive response placed the mother in the “*Use of formal childcare*” category, while a negative response led to another question: “*Would you have preferred to send the child to daycare?*” If the answer was positive, the mother was categorised under “*Suboptimal choice.*”

Mothers in the “*Suboptimal choice*” and “*No one to ask*” categories represent, in our interpretation, two distinct aspects of the unmet need for formal childcare. The former group relies on informal childcare but would have preferred to use formal childcare services. In contrast, the latter group consists of mothers who lack access to both formal and informal childcare options and, as a result, must personally take care of their children due to an unaddressed need for support.

We operationalised the migrant background variable in three distinct dimensions. First, we used a dichotomous variable which identifies the “migrant status” distinguishing between native and migrant mothers based on place of birth.

Additionally, we made one-step forward to overcome the native-migrant dichotomy and further considered the “migrant generation”. We distinguished the following groups: 0 “Natives”, 1 “1st-generation”, and 2 “1.5-generation” migrants. We merged the group of second generations with the Italians for analytical purposes due to a limited number of respondents in the former group (*n* = 18). In our classification, a woman migrating at the age of 18 or younger falls into the 1.5-generation group, while those migrating after reaching age 18 are categorised as 1st-generation. All other mothers in the study who did not fall into these migration categories were considered as natives. Furthermore, eight respondents were excluded from the sample due to missing information concerning their age at migration. About 13% are migrant mothers, among them, 77% are 1st-generation migrants, while 23% are 1.5-generation migrants^3^ (Tables 3 and 4 in the Appendix).

Finally, to achieve a comprehensive understanding of how migration processes influence diverse family structures, the last dimension of the migration background refers to the combination of the parents' migration background. To this end, we combined variables related to the migratory background of the mother (native; migrant) and that of the father by whom she had the child she was interviewed for (native; migrant). Therefore, the combination of parents' migration background variable includes the following four categories: (1) “both native parents (84%)”, (2) “native mother and migrant father (3%)”, (3) “migrant mother and native father (4%)”, and (4) “both migrant parents (9%)”. Tables 3 and 4 in the Appendix show the distribution of the dependent and the “Use of formal childcare” variables across migration background.

### Control Variables

In all analyses, we control for the socio-economic and demographic variables discussed earlier in the paper. Age at interview (“  ≤ 24”, 25–29”, “30–34”, “35–39”, and “40 and over”); Parity at interview (“1”, “2”, and “3 or more”); macro-area of residence (“North”, “Centre”, and “South”); and mother's level of education (“primary”, “secondary”, and “tertiary”).

Detailed descriptive statistics for all variables used in the models across the dependent variable categories are provided in Table 2 in the Appendix.

## Method and Analytical Strategy

We use the multinomial logistic regression model due to its suitability for analysing our dependent variable with multiple categories and the absence of proportional odds. This approach allows us to independently estimate relationships between our independent variables and each category of the dependent variable separately, without imposing the constraints of the ordinal logistic model.

To model the association between the use of (or unmet need for) formal childcare and fertility intentions and behaviour, we conducted four separate analyses. First, we assessed the overall relationship between formal childcare use and fertility intentions and outcomes. Second, we explored how this association varies by migratory background. Third, we differentiated by migrant generation, and fourth, by parental migratory background.

We computed relative risk ratios from each model, and subsequently, to enhance the readability of results, we computed predicted probabilities of the outcomes with confidence intervals centred on the predictions and with lengths equal to 2 × 1.39 × standard errors. This was necessary to obtain an average level of 5% for Type I errors in pairwise comparisons of a group of means (Goldstein & Healy, 1995). The full models are shown in the Appendix (Tables 6, 7, and 8).

## Results

### Formal Childcare Use and Fertility

**Fig. 2:**
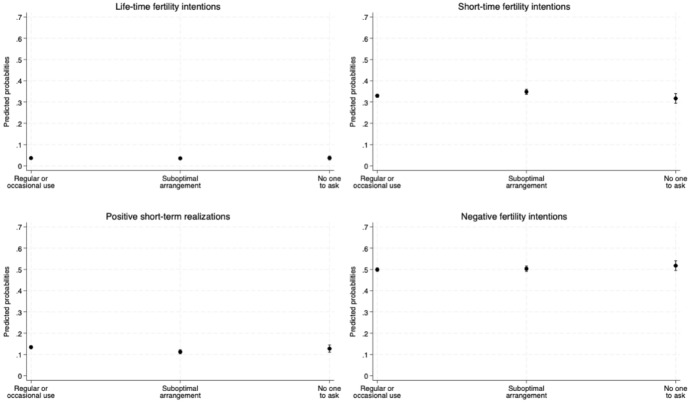
Adjusted predicted probabilities of having positive and negative fertility intentions and behaviours by formal childcare use. *Notes*: Results from the multinomial logistic regression. The model controls for age at interview, parity at interview, area of residence, mother's level of education, and migrant background. Control variables at mean value. 83.5%. CI. The model is weighted. *Source*: Authors' elaboration on Birth Sample Survey/Indagine campionaria sulle nascite e le madri (2012)

Figure 2 illustrates the predicted probabilities of positive (both lifetime and short-term) and negative fertility intentions, as well as positive short-term realisations, based on the mother's use of formal childcare for the whole sample.

Our findings indicate that the use of formal childcare has a relatively negligible influence on the likelihood of having positive fertility intentions. However, mothers who report having “no one to ask” show a slightly higher probability of expressing negative fertility intentions (52%) compared to those using formal childcare or a suboptimal arrangement (50%). Additionally, mothers who use formal childcare demonstrate slightly higher probabilities of being pregnant or already having another child (approximately 13%) compared to those who do not use formal childcare (approximately 11%).

### The Role of Migration Background

#### Differences Between Native and Migrant Mothers

**Fig. 3:**
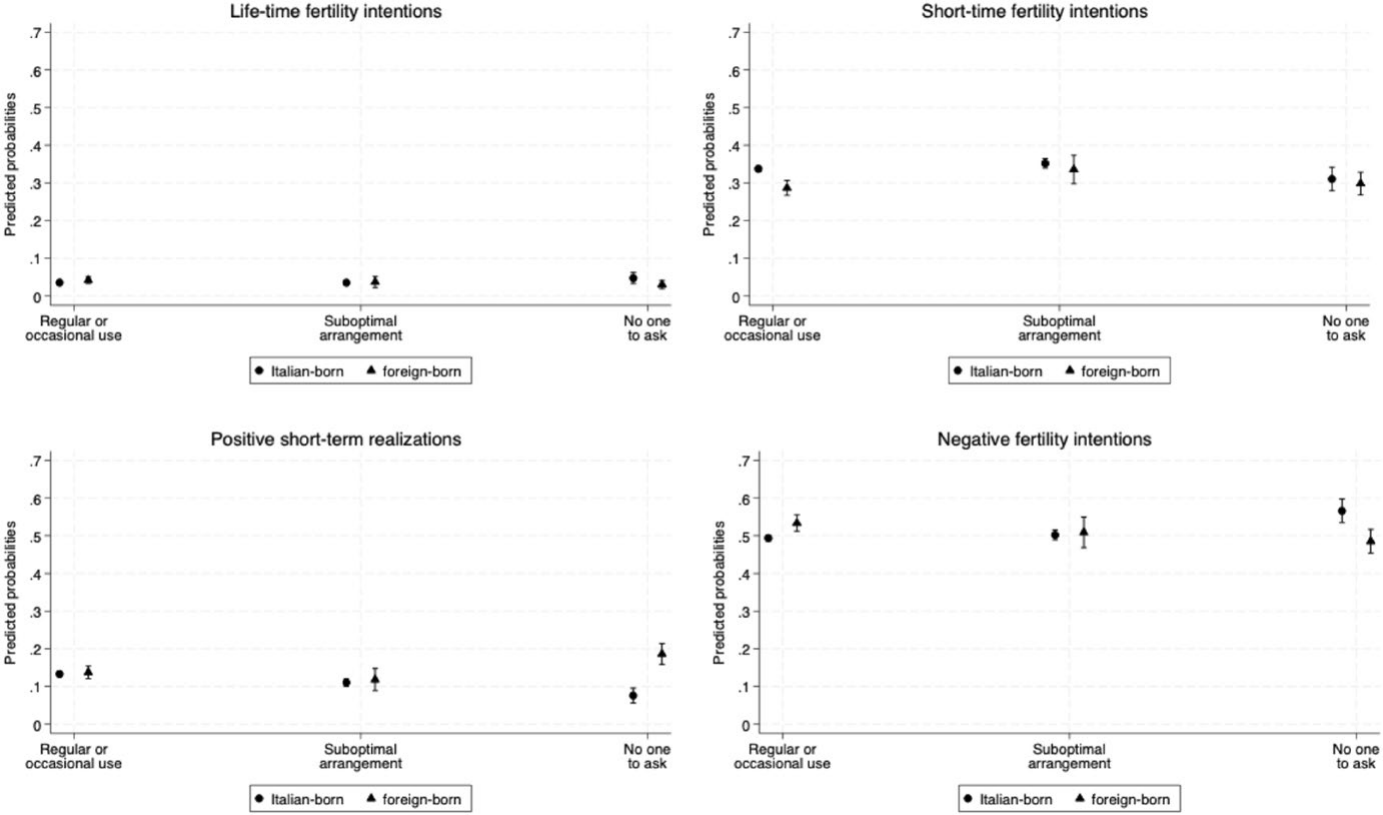
Adjusted predicted probabilities of having positive and negative fertility intentions and behaviours by formal childcare use for both native and migrant mothers. *Notes*: Results from the multinomial logistic regression. The model controls for age at interview, parity at interview, area of residence, and mother's level of education. Control variables at mean value. Interaction term with mother' use of formal childcare and migrant status added. 83.5% CI. The model is weighted. *Source*: Authors' elaboration on Birth Sample Survey/Indagine campionaria sulle nascite e le madri (2012)

To investigate our second aim, we examined whether there are differences in the link between mothers' use of formal childcare and their fertility intentions and behaviours by migration background. Here, we categorised mothers into two groups: Italian-born and foreign-born mothers. Results are shown in Fig. 3. Lifetime fertility intentions do not reveal any particular pattern. As for short-term fertility intentions, Italian-born mothers using formal childcare exhibit a higher probability of expressing positive intentions (about 34%) compared to foreign-born mothers (about 29%). Interestingly, this pattern persists even among mothers who do not use formal childcare, albeit in this case, differences are not statistically significant. Italian-born mothers who report having no one to ask for help demonstrate a higher likelihood of expressing negative intentions (about 57%) compared to their foreign-born counterparts (about 48%). Finally, the relationship between formal childcare use and fertility behaviours differs markedly between Italian-born and foreign-born mothers. Among Italian-born mothers, the use of formal childcare corresponds to the highest probability of being pregnant or having an additional child (about 13%), while reporting no one to ask is associated with the lowest probability (about 7%). In contrast, among foreign-born mothers, the highest probability of being pregnant or having an additional child is observed among those who report having no one to ask for help (about 18%), compared to approximately 12% for mothers in the other childcare categories.

#### Differences by Migrant Generation

**Fig. 4:**
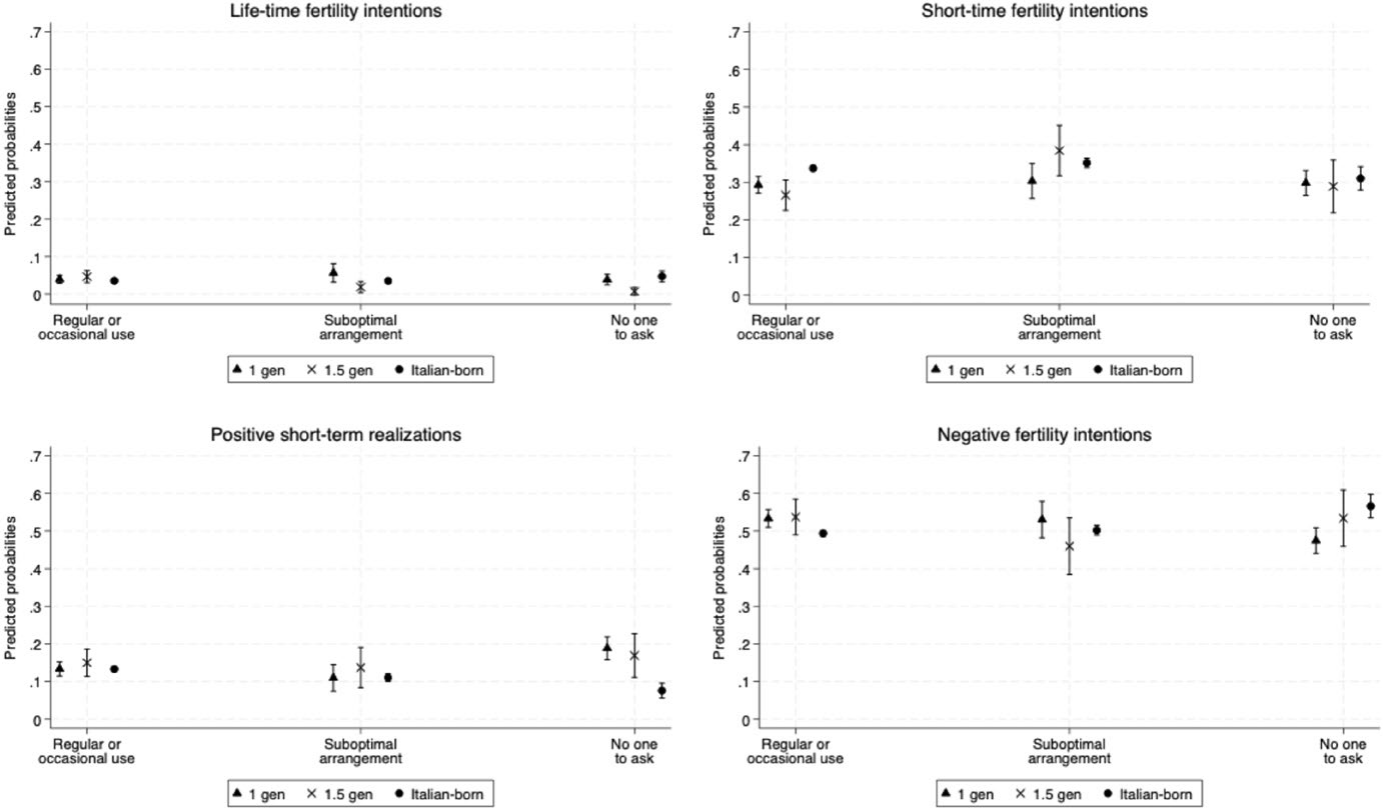
Adjusted predicted probabilities of having positive and negative fertility intentions and behaviours by formal childcare use and migrant generation. *Notes*: Results from the multinomial logistic regression. The model controls for age at interview, parity at interview, area of residence, mother's level of education, and economic situation. Control variables at mean value. Interaction term with childcare use and migrant generation added. 83.5% CI. The model is weighted. Full models are not shown in this paper but are available upon request. *Source*: Authors' elaboration on Birth Sample Survey/Indagine campionaria sulle nascite e le madri (2012)

Figure 4 displays predicted probabilities of positive and negative fertility intentions and positive fertility behaviours by formal childcare use and migrant generation.

An interesting finding is the heterogeneity observed among women with a migrant background when distinguishing between 1st- and 1.5-generation migrants. Specifically, 1st-generation mothers who report having no one to ask for support exhibit a higher likelihood of positive lifetime fertility intentions compared to 1.5-generation mothers, as well as a lower likelihood of negative fertility intentions. Conversely, among those with suboptimal childcare arrangements, 1.5-generation mothers report higher probabilities of short-term fertility intentions and realisations, along with lower probabilities of negative fertility intentions, compared to 1st-generation mothers, albeit these differences are not statistically significant.

#### Differences by Parents' Migratory Background

**Fig. 5:**
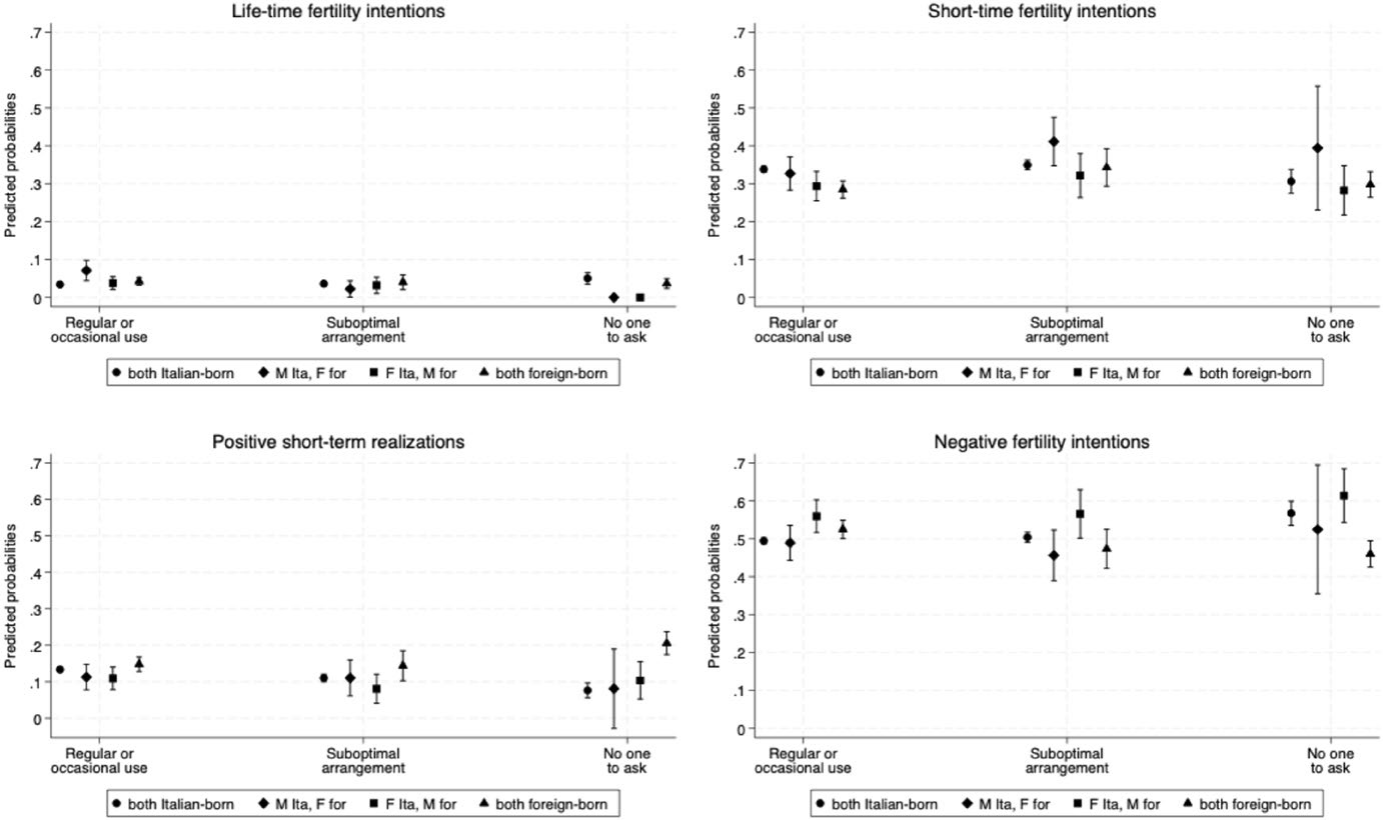
Adjusted predicted probabilities of having positive and negative fertility intentions and behaviours by formal childcare use and parents' background. *Notes*: Results from the multinomial logistic regression. Model controls for age at interview, parity at interview, area of residence, and mother's level of education. Control variables at mean value. Interaction term with mother's use of formal childcare and parents' background added. 83.5% CI. The model is weighted. Full models are not shown in this paper but are available upon request. *Source*: Authors' elaboration on Birth Sample Survey/Indagine campionaria sulle nascite e le madri (2012)

**Fig. 6:**
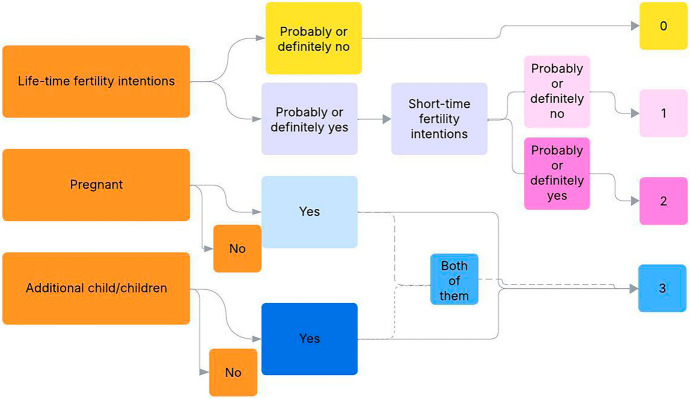
Survey question flow for the variable “fertility intentions and behaviours”. Data: Birth Sample Survey/Indagine campionaria sulle nascite e le madri 2012.* Source*: Own elaboration

Figure 5 displays the predicted probabilities of having positive and negative fertility intentions and behaviours by parents' background—comprising both parents are Italian-born, both are foreign-born, or mixed couples with either a native mother and a migrant father, or vice-versa.

Among mixed couples, having no one to ask for support is associated with a lower likelihood of positive lifetime fertility intentions compared to both couples using formal childcare and couples in which both parents are either Italian-born or foreign-born. Among couples with Italian-born man and foreign-born woman, the predicted probability of positive short-term fertility intentions is especially high when they have no one to ask or in case of suboptimal arrangements (about 40%); however, confidence intervals are quite large due to the reduced sample size, and the difference is not significant. Couples where both parents are Italian-born report the lowest probabilities of short-term fertility intentions and realisations and the highest probabilities of negative fertility intentions when they have no one to ask for support.

Couples where both parents are migrants demonstrate the highest likelihood of positive short-term realisations and lowest negative fertility intentions when they report having no one to ask for support (20%, compared to 7% for both Italians, 10% for migrant mothers and Italian father, and 8% for Italian mothers and migrant father).

### The Role of Control Variables

In addition to our key variables of interest, the models incorporate the control variables outlined in the background section. Although not the primary focus, their relationships with our outcome of interest are briefly discussed in the following paragraph. (Full models are reported in Tables 6 and 9 in the Appendix). As expected, parity is negatively associated with all fertility outcomes: Mothers with two or more children are less likely to express positive intentions or have additional births. Similarly, age at interview shows a negative association, with older women being less likely to report positive fertility intentions or having additional children. Mothers' educational attainment tends to be positively associated with fertility outcomes, with tertiary-educated mothers showing significantly higher likelihoods of both intentions and behaviours—particularly for short-term intentions and being pregnant or with another child.

Regarding geographical controls, while macro-area of residence does not significantly relate to most outcomes, living in the Centre is significantly associated with a lower likelihood of being pregnant or already with another child. In the stratified models by parity, we observe that among mothers with two or more children, residing in the Centre or South is associated with significantly lower short-term fertility intentions compared to those residing in the North. This pattern aligns with more recent findings in Italian demography, which highlight comparatively lower fertility levels in the South among both native and migrant populations.

## Robustness Checks

We performed comprehensive robustness checks to ensure the reliability of our results and the consistency of our findings across different model specifications.

We conducted a first check using a binary dependent variable: (1) respondents who expressed a desire for a child, planned to have one soon, were already pregnant, or had given birth to a child after the one they were interviewed for, and (2) those who indicated they did not want another child. The results of this model confirm the validity of our main findings (full models are available upon request). Ultimately, we chose the four-category variable as the primary outcome measure, as this approach recognises that fertility intentions and behaviour, as well as short-term and lifetime intentions, represent distinct groups influenced by different factors. By employing this nuanced classification, we can better capture the immediacy and specificity of fertility intentions within a 3-year timeframe.

Additionally, a key control variable in our models is the mother's level of education. Due to the potential collinearity with employment status and economic situation, we opted not to include employment status and economic situation directly in the main model. However, to test the robustness of our results, we ran three additional models: one including “economic situation”, another one including “respondent's employment status”, and a third one accounting for “couple's employment status”. These variables were not statistically significant, and when they were, they masked the effect of education.

Furthermore, we ran parity-specific models, presented in Table 9 in the Appendix. Despite some estimates losing significance in the group of women with one child, results prove that the link between migrant background, childcare use, and fertility intentions/behaviours is substantially similar among women with one child and women with more than one child.

## Discussion and Conclusion

This is the first study to investigate the relationship between mothers' use of formal childcare services and their fertility intentions and behaviours by migrant background using nationally representative data in Italy. By comparing perspectives from both native and migrant mothers in Italy, we aim to uncover how the use of—or the unmet need for—formal childcare affects fertility dynamics across different population groups.

Addressing our primary question, “*Does utilizing childcare services for one child positively affect mothers' inclinations to have another?,*” our findings support our first hypothesis (H1). Mothers who regularly or occasionally use formal childcare for a prior child tend to exhibit more positive fertility behaviours compared to those not relying on formal childcare due to a suboptimal arrangement or to the impossibility to ask someone. However, this relationship is observed mainly in fertility behaviours rather than intentions, partially confirming our hypothesis (H2). Nonetheless, the differences observed between formal childcare users and non-users were minimal, echoing existing literature that suggests modest effects of family policies on fertility (e.g. Brainerd, 2014; Guetto et al., 2025; Hank & Kreyenfeld, 2003; Scherer et al., 2023). This underscores the challenges in policy implementation and the complexity inherent in fertility decisions, aligning with the prevailing view that, although crucial, family policies alone are unlikely to markedly change fertility trends.

In comparing native and migrant populations, consistent with our hypothesis (H3), the use of formal childcare services has a differential effect on the two groups. We expected that Italian-born mothers, who rely more on formal childcare services, would be more incentivised to intend to have and have another child (H3a), and our findings generally support this expectation. In contrast, foreign-born mothers tend to have another child regardless of their use of formal childcare services for the previous birth. We also posited, though, that given the multiple challenges faced by migrant mothers in the host country, unmet childcare needs could be a powerful deterrent to fertility for them (H3b). Contrary to our hypothesis, unmet childcare needs (especially having no one to ask for support) negatively affect natives more than migrants.

We might speculate about the underlying causes of these findings by referencing prior research and examining data from national statistics. On the one hand, in some migrant communities in Italy, such as those from Northern Africa, Sub-Saharan Africa, parts of Asia, and Eastern Europe, fertility may be less “planned”, with a greater reliance on divine will or fate rather than the pursuit of optimal conditions (Milewski & Mussino, 2018). Additionally, migrant mothers frequently adhere to traditional gender roles (with wide heterogeneity by origin), often staying out of the labour market and primarily responsible for childcare (Trappolini et al., 2023), reducing their reliance on formal childcare services.

Moreover, respondents with migrant backgrounds often face challenges re-entering the labour market after leaving, as highlighted in the literature (Kil et al., 2018). Having recently had a first child might have prompted our respondents to use that window for a second child, regardless of their childcare options. Our findings offer partial support for the notion that *1st-generation* migrant mothers are less responsive to formal childcare services than 1.5-generation mothers (H4), which is broadly consistent with the adaptation framework widely discussed in migration research (e.g. Kulu, 2005; Andersson et al., 2004; Milewski, 2010). According to this hypothesis, migrants' behaviour converges towards that of the host population across generations due to increased exposure to local norms and institutions (Milewski, 2007). In line with adaptation arguments, our results suggest that 1.5-generation mothers, having spent more of their childhood in Italy, are more inclined to rely on institutional childcare. In contrast, *1st-generation* mothers appear to maintain stronger reliance on family networks—consistent with socialisation perspectives—especially when facing childcare constraints. These findings also resonate with recent evidence indicating that *1st-generation* migrants may have limited access to local daycare systems and instead turn to extended family for support (Mussino & Ortensi, 2023). Additionally, we examined how two unmet needs scenarios differently affect the respondents. *1st-generation* mothers exhibited higher fertility realisations when lacking childcare support (“no one to ask”), whereas 1.5-generation mothers had higher fertility intentions when facing suboptimal childcare arrangements.

Regarding our last hypothesis (H5), which suggested that mothers in mixed couples would exhibit a higher positive impact from the use of formal childcare on fertility than couples with two migrant parents but less than couples with two Italian parents, we found partial confirmation. Specifically, patterns varied by parental background: Couples with foreign-born mothers and Italian-born fathers show a higher likelihood of positive lifetime fertility intentions when regularly using formal childcare. Conversely, couples with both members foreign-born exhibit the most positive fertility behaviours even without formal childcare (due to suboptimal choice or impossibility to ask someone), indicating that alternative family care support, such as parental care or grandparents and other family member care mechanisms, may play a role.

Our findings show a nuanced and multifaceted picture, underscoring the need for comprehensive institutional support. While the significance of formal childcare services in enabling parental, particularly maternal, workforce participation, promoting gender equality, supporting the well-being of young generations and fostering a more equitable society is undeniable (Bousselin, 2022), our findings suggest that these services alone may not be sufficient to significantly change fertility patterns. However, the outcome measure used in this study is not ideal for assessing long-term fertility. This is because we are observing behaviours within a limited timeframe, and it is known that the link between intentions and long-term fertility is weaker.

Nonetheless, addressing the unmet childcare needs of both native and migrant mothers remains imperative for fostering gender equality, driving economic growth, as well as assisting families in achieving their fertility intentions. The significance of this issue is particularly pronounced in the Italian context, where disparities in domestic and caregiving responsibilities persist and the lack of adequate childcare options disproportionately affects women, hindering their full workforce participation (Mussida & Patimo, 2021). Therefore, ensuring the fulfilment of care needs through high-quality and accessible childcare services for all is a key strategy to mitigate these challenges and to enable parents to balance work and family life more effectively, thereby empowering them to make more autonomous decisions regarding their fertility.

Our contribution to the literature is threefold. First, we adopted a comprehensive approach by examining both fertility intentions and behaviours (even if in the short term). Second, this is the first study that evaluates the varying effects of unmet need for childcare on mothers from both native and migrant backgrounds in Italy. Third, in exploring migration backgrounds, we first used a dichotomous variable to distinguish between native-born mothers and those born abroad. However, to provide a more comprehensive understanding, we included variables for migrant generations and parents’ background.

Nevertheless, the study presents some limitations that must be taken into account when interpreting the results. The primary issue is the dated nature of the Birth Sample Survey, collected in 2012. Although the results pertaining to 2012, they remain still reliable for two reasons. First, over the past decade, family policies in Italy have seen minimal change. For instance, from 2012 to 2019, there was no substantial increase in the provision of early childhood services, leaving Italy at the bottom of the European Union rankings (ISTAT, 2019b; Naldini & Saraceno, 2022). In 2022, a unified child allowance system was implemented, consolidating various fragmented and disparate measures, but its effects are still not measurable. Second, in the past decade, there has been an increase in the settlement of the first generation of migrants, primarily through family reunification with children (Trappolini et al., 2023). Nevertheless, more recent data would offer better insights into existing childcare patterns and a more up-to-date representation of the Italian migration panorama.

We do not have specific information on migrants' countries of birth (i.e. we only know if individuals are born abroad); the survey only collects their last country of residence before moving to Italy. This precludes the performing of country of birth-specific analyses. Given the significant differences across nationalities, we hypothesise that the impact of childcare on fertility would vary when controlled for the ethnic background.

Additionally, the survey exclusively gathers responses from mothers and does not include information on the perspectives of partners. Nevertheless, consistent with existing literature (Berrington, 2004), we interpret the collected data as resulting from intra-couple deliberation, thereby reflecting men's intentions as well.

Some limitations may arise in interpreting the items within the questionnaire due to the lack of detailed explanations that would enable a clearer understanding of what they refer to or how parents have perceived them. Consequently, we have opted for our interpretation, which inherently carries a degree of subjectivity. Specifically, we have chosen to classify those who declared having no one to ask for childcare under “unmet need”, aligning with Mussino and Ortensi (2023). We interpreted a mother's declaration of having no one to ask for childcare as indicating both an unawareness of how to access formal childcare services and the absence of an informal network to rely on for her child's care. Another critical issue arises from the interpretation of a question posed to mothers who did not use formal childcare services as their primary choice of external childcare but instead relied on alternative forms of childcare: “Do you occasionally use formal childcare services?” A positive answer placed the mother in the “Use of formal childcare” category for using formal childcare. Yet, we recognise that various motivations or circumstances might lead to this choice. This categorisation encompasses a spectrum of childcare use, reflecting both preference and necessity. On the one hand, some might opt for formal childcare due to a preference for informal childcare options—such as relatives, friends, or neighbours—or other paid options, such as a childminder, and only turn to formal childcare as needed. Others, instead, might like to utilise formal childcare more frequently but could be constrained by several factors. These limitations can range from logistical issues, such as the distance to the childcare facility, to financial constraints, or even due to eventual experiences of illness, particularly frequent in group settings like daycare.

Despite all these limitations, our results yield valuable insights into the intricate relationship between formal childcare use and fertility intentions and behaviours among both native and migrant mothers in Italy. While the solely use of formal childcare appears to slightly influence only (short term) fertility behaviours, the significance of unmet childcare needs emerges as a critical factor, affecting mothers' fertility trajectories differently across population groups. These findings highlight the complexity of fertility decision-making and the multifaceted influences shaping individuals' reproductive choices. As such, our study underscores the importance of comprehensive policy approaches that address not only the availability and accessibility of formal childcare services but also the broader socio-economic and cultural factors impacting fertility decisions.

## Appendix

See Tables 2, 3, 4, 5, 6, 7, 8, 9 and Fig. 6.

**Table 2:** Distribution of the independent and control variables across the categories of the dependent variable

% weighted	Negative intentions	Positive lifetime intentions	Positive short-time intentions	Positive short-time realisations
*Childcare use (main specification)*				
Use of formal childcare	49.6%	3.7%	33.6%	13.1%
Suboptimal arrangement	49.6%	3.7%	35.7%	11.0%
No one to ask	53.2%	5.2%	25.3%	16.3%
*Migrant background*				
Foreign-born	42.9%	4.5%	31.9%	20.7%
Italian-born	53.1%	3.7%	32.6%	10.6%
Natives	53.1%	3.7%	32.6%	10.6%
*Parents’ migrant background*				
Both partners natives	53.4%	3.6%	32.4%	10.6%
Mother native/Father migrant	44.6%	5.3%	38.9%	11.2%
Mother migrant /Father native	49.8%	3.8%	34.6%	11.8%
Both foreign-born	40.7%	4.7%	31.1%	23.5%
*Age*				
Until 24	16.7%	17.6%	46.4%	19.3%
25–29	26.5%	7.6%	45.7%	20.2%
30–34	42.9%	3.4%	39.4%	14.3%
35–39	63.3%	1.5%	25.9%	9.3%
40 and more	81.9%	0.5%	13.4%	4.3%
*Parity at interview*				
1	22.5%	5.9%	61.8%	9.9%
2	70.6%	2.7%	12.5%	14.3%
3 or more	79.0%	1.1%	5.7%	14.2%
*Grandparents cohabiting*				
Yes	37.6%	9.5%	41.4%	11.5%
No	52.2%	3.4%	31.9%	12.5%
*Area*				
North	51.9%	3.0%	31.6%	13.5%
Central	50.0%	4.0%	33.9%	12.2%
South	51.1%	4.9%	33.1%	11.0%
*Educational level*				
Primary	58.6%	4.4%	26.4%	10.6%
Secondary	49.9%	4.1%	34.2%	11.8%
Tertiary	43.8%	2.4%	37.6%	16.3%
*Couple's employment status*				
No earner	47.3%	7.2%	30.9%	14.5%
Single earner couple	51.9%	4.5%	29.7%	14.0%
Dual earner couple	51.2%	2.9%	35.1%	10.8%
*Income spent monthly*				
Less of about half	44.5%	3.4%	38.1%	13.9%
More than half	52.1%	3.1%	33.6%	11.2%
All	54.2%	4.8%	27.7%	13.4%
*Childcare use (alternative specification)*				
Formal care	50.5%	3.8%	33.0%	12.7%
Occasionally	44.6%	2.7%	37.8%	15.0%
No, by choice	52.4%	3.8%	31.9%	11.9%
Unmet need	50.7%	4.1%	32.5%	12.6%
Share of observations	51.3%	3.8%	32.5%	12.4%
*N* (Number of observations)	9263	712	6071	2275

Data: Birth Sample Survey/Indagine campionaria sulle nascite e le madri 2012. Percentages are weighted and should be read by row. *Source*: Own elaboration

**Table 3:** Distribution of the dependent and the explanatory variables by migratory background

% weighted	Natives	1st-generation	1.5-generation
*Fertility behaviours*			
Negative	53.1%	44.1%	38.5%
Positive lifetime intentions	3.7%	3.7%	7.3%
Positive short-time intentions	32.6%	31.9%	31.8%
Positive short-term realisations	10.6%	20.3%	22.5%
*Childcare use (alternative specification)*			
Formal care	25.2%	27.3%	25.8%
Occasionally	4.5%	3.1%	2.7%
No, by choice	56.3%	46.2%	47.8%
Unmet need	13.9%	23.5%	23.7%
*Childcare use (main specification)*			
Use of formal childcare	68.1%	56.3%	54.6%
Suboptimal arrangement	26.1%	13.7%	24.3%
No one to ask	5.8%	30.0%	21.1%
*N* (Number of observations)	15901	1866	546

Data: Birth Sample Survey/Indagine campionaria sulle nascite e le madri 2012. Percentages are weighted and should be read by column. *Source*: Own elaboration

**Table 4:** Distribution of the dependent and independent variables by migratory background

% weighted	Foreign-born	Italian-born
*Migrant generation*		
1st-generation	79.0%	0.0%
1.5-generation	21.0%	0.0%
Natives	0.0%	100%
*Parents’ migrant background*		
Both partners natives	0.0%	97.0%
Mother native/Father migrant	0.0%	3.0%
Mother migrant/Father native	23.7%	0.0%
Both partners migrants	76.3%	0.0%
*Age*		
Until 24	10.8%	4.9%
25–29	29.3%	12.5%
30–34	31.5%	30.5%
35–39	21.0%	36.0%
40 and more	7.5%	16.1%
*Parity at interview*		
1	43.7%	42.5%
2	38.2%	43.3%
3	18.1%	14.2%
*Grandparents cohabiting*		
Yes	10.8%	5.6%
No	89.2%	94.4%
*Area*		
North	67.2%	43.0%
Central	20.9%	17.2%
South	11.9%	39.8%
*Educational level*		
Primary	32.3%	29.6%
Secondary	45.2%	50.2%
Tertiary	22.5%	20.2%
*Couple's employment status*		
No earner	9.4%	4.4%
Single earner couple	62.9%	40.0%
Dual earner couple	27.7%	55.6%
*Income spent monthly*		
Less of about half	26.0%	24.3%
More than half	31.5%	44.9%
All	42.5%	30.8%
*Fertility behaviours*		
Negative	42.9%	53.1%
Positive lifetime intentions	4.5%	3.7%
Positive short-time intentions	31.9%	32.6%
Positive short-term realisations	20.7%	10.6%
*Childcare use*		
Use of formal childcare	55.9%	68.1%
Suboptimal arrangement	15.9%	26.1%
No one to ask	28.2%	5.8%
*N* (Number of observations)	2420	15901

Data: Birth Sample Survey/Indagine campionaria sulle nascite e le madri 2012. Percentages are weighted and should be read by column. *Source*: Own elaboration

**Table 5:** Distribution of the dependent and independent variables across the categories of the childcare use variable

% weighted	Childcare use regular	Childcare use occasional	Childcare NO, by choice	Childcare—unmet need
*Migrant background*				
Foreign-born	26.9%	3.0%	46.5%	23.6%
Italian-born	25.4%	4.5%	56.2%	13.9%
*Migrant generations*				
1st-generation	27.2%	3.0%	46.2%	23.6%
1.5-generation	25.8%	2.8%	47.7%	23.7%
Natives	25.4%	4.5%	56.2%	13.9%
*Parents’ migrant background*				
Both partners natives	25.3%	4.5%	56.2%	14.0%
Mother native/Father migrant	23.0%	4.7%	60.4%	11.9%
Mother migrant /Father native	22.3%	2.6%	55.8%	19.3%
Both partners migrants	28.3%	3.1%	43.7%	24.9%

Data: Birth Sample Survey/Indagine campionaria sulle nascite e le madri 2012. Percentages are weighted and should be read by row. *Source*: Own elaboration

**Table 6:** Multinomial regression on fertility intentions and behaviours: relative risk ratios (reference negative fertility intentions)

Life-term positive intentions	Model 1	Model 1c	Model 2	Model 2c
*Childcare use* *(regular* + *occasionally)*				
Suboptimal arrangements	0.93	0.97	0.94	0.98
No one to ask	0.87	0.94	0.95	1.04
*Migrant background (Italian-born)*				
Foreign-born	0.89	0.95	0.92	0.99
*Age (Until 24)*				
25–29	0.41***	0.38***	0.41***	0.38***
30–34	0.12***	0.11***	0.12***	0.11***
35–39	0.04***	0.04***	0.04***	0.04***
40 and more	0.01***	0.01***	0.01***	0.01***
*Parity (1)*				
2	0.25***	0.25***	0.25***	0.25***
3 and more	0.11***	0.12***	0.11***	0.12***
*Childcare use#migratory* *(regular* + *occasionally #Foreign-born)*				
Suboptimal arrangements #Foreign-born			1.01	1.02
No one to ask #Foreign-born			0.82	0.79
*Area (North)*				
Central		1.05		1.05
South		1.00		0.99
*Education (Primary)*				
Secondary		1.61**		1.62***
Tertiary		1.43		1.44

Short-term intentions	Model 1	Model 1c	Model 2	Model 2c
*Childcare use* *(regular* + *occasionally)*				
Suboptimal arrangements	1.02	1.07	0.98	1.04
No one to ask	0.81	0.89	0.64**	0.71*
*Migrant background (Italian-born)*				
Foreign-born	0.75***	0.80**	0.64***	0.69**
*Age (Until 24)*				
25–29	0.90	0.83	0.89	0.82
30–34	0.61***	0.52***	0.60**	0.52***
35–39	0.32***	0.27***	0.32***	0.27***
40 and more	0.11***	0.09***	0.11***	0.09***
*Parity (1)*				
2	0.07***	0.07***	0.07***	0.07***
3 and more	0.04***	0.04***	0.039***	0.04***
*Childcare use#migratory* *(regular* + *occasionally #Foreign-born)*				
Suboptimal arrangements #Foreign-born			1.33	1.31
No one to ask #Foreign-born			1.80*	1.71*
*Area (North)*				
Central		0.88		0.88
South		0.99		0.99
*Education (Primary)*				
Secondary		1.23		1.23*
Tertiary		1.66***		1.64***

Pregnant or with child	Model 1	Model 1c	Model 2	Model 2c
*Childcare use* *(regular* + *occasionally)*				
Suboptimal arrangements	0.75***	0.83*	0.73***	0.81*
No one to ask	0.76	0.89	0.37***	0.46***
*Migrant background (Italian-born)*				
Foreign-born	0.99	1.13	0.76*	0.90
*Age (Until 24)*				
25–29	0.96	0.80	0.93	0.78
30–34	0.52***	0.37***	0.5***	0.36***
35–39	0.22***	0.15***	0.21***	0.15***
40 and more	0.05***	0.038***	0.05***	0.03***
*Parity (1)*				
2	0.53***	0.55***	0.53***	0.55***
3 and more	0.70***	0.78*	0.70***	0.78*
*Childcare use#migratory* *(regular* + *occasionally #Foreign-born)*				
Suboptimal arrangements #Foreign-born			1.21	1.15
No one to ask #Foreign-born			3.84***	3.42***
*Area (North)*				
Central		0.82*		0.82*
South		1.01		1.04
*Education (Primary)*				
Secondary		1.31*		1.30*
Tertiary		2.67***		2.62***

Independent variables: childcare use and migrant background (Italian-born/foreign-born)

Data: Birth Sample Survey/Indagine campionaria sulle nascite e le madri 2012

Note: **p* < 0.05; ***p* < 0.01; and ****p* < 0.001

Model 1 is the baseline model without control variables or interactions. Model 1c includes the interaction term between migrant background and childcare use. Model 2 introduces control variables but excludes interaction effects, while Model 2c includes both control variables and the interaction term

*Source*: Own elaboration

**Table 7:** Multinomial regression on fertility intentions and behaviours: relative risk ratios (reference negative fertility intentions)

Life-term positive intentions	Model 2c
*Childcare use* *(regular* + *occasionally)*	
Suboptimal arrangements	0.98
No one to ask	1.04
*Migrant background (Native)*	
1st-generation	0.93
1.5-generation	1.06
*Childcare use#migratory* *(regular* + *occasionally #1gen)*	
Suboptimal arrangements #1gen	1.54
Suboptimal arrangements #1.5gen	0.57
No one to ask #1gen	1.16
No one to ask #1.5gen	0.14

Short-term intentions	
*Childcare use* *(regular* + *occasionally)*	
Suboptimal arrangements	1.04
No one to ask	0.71*
*Migrant background (Native)*	
1st-generation	0.72**
1.5-generation	0.62*
*Childcare use#migratory* *(regular* + *occasionally #gen)*	
Suboptimal arrangements #1gen	1.04
Suboptimal arrangements #1.5gen	2.08
No one to ask #1gen	1.73*
No one to ask #1.5gen	1.52

Pregnant or with child	
*Childcare use* *(regular* + *occasionally)*	
Suboptimal arrangements	0.81*
No one to ask	0.46***
*Migrant background (Native)*	
1st-generation	0.88
1.5-generation	0.97
*Childcare use#migratory* *(regular* + *occasionally #gen)*	
Suboptimal arrangements #1gen	1.03
Suboptimal arrangements #1.5gen	1.45
No one to ask #1gen	3.70***
No one to ask #1.5gen	2.45*

Independent variables: childcare use and migrant generation (Native, 1st-generation, and 1.5-generation)

Data: Birth Sample Survey/Indagine campionaria sulle nascite e le madri 2012

Note: **p* < 0.05; ***p* < 0.01; and ****p* < 0.001

Only Model 2c is presented, which includes control variables and the interaction term between migrant generation and childcare use. The coefficients for control variables are not reported, as they are similar to those in Table A5

*Source*: Own elaboration

**Table 8:** Multinomial regression on fertility intentions and behaviours: relative risk ratios (reference negative fertility intentions)

Life-term positive intentions	Model 2c
*Childcare use* *(regular* + *occasionally)*	
Suboptimal arrangements	1.04
No one to ask	1.15
*Migrant background (Both natives)*	
Mother native/Father migrant	2.23*
Mother migrant /Father native	0.85
Both migrants	1.07
*Childcare use#migratory* *(regular* + *occasionally #gen)*	
Suboptimal arrangements #M ita/F for	0.34
Suboptimal arrangements #F ita/M for	0.81
Suboptimal arrangements #both For	1.15
No one to ask #M ita/F for	3.03
No one to ask #F ita/M for	5.12
No one to ask #both For	0.95

Short-term intentions	
*Childcare use* *(regular* + *occasionally)*	
Suboptimal arrangements	1.03
No one to ask	0.70*
*Migrant background (Both natives)*	
Mother native/Father migrant	1.01
Mother migrant /Father native	0.66*
Both migrants	0.71**
*Childcare use#migratory* *(regular* + *occasionally #gen)*	
Suboptimal arrangements #M ita/F for	1.48
Suboptimal arrangements #F ita/M for	1.09
Suboptimal arrangements #both For	1.52
No one to ask #M ita/F for	1.57
No one to ask #F ita/M for	1.09
No one to ask #both For	1.88*

Pregnant or with child	
*Childcare use* *(regular* + *occasionally)*	
Suboptimal arrangements	0.81*
No one to ask	0.46***
*Migrant background (Both natives)*	
Mother native/Father migrant	0.86
Mother migrant /Father native	0.67
Both migrants	1.00
*Childcare use#migratory* *(regular* + *occasionally #gen)*	
Suboptimal arrangements #M ita/F for	1.35
Suboptimal arrangements #F ita/M for	0.90
Suboptimal arrangements #both For	1.43
No one to ask #M ita/F for	1.37
No one to ask #F ita/M for	1.74
No one to ask #both For	3.70***

Independent variables: childcare use and parents’ migration background

Data: Birth Sample Survey/Indagine campionaria sulle nascite e le madri 2012

Note: **p* < 0.05; ***p* < 0.01; and ****p* < 0.001

Only Model 2c is shown, as it incorporates control variables. The coefficients for control variables are not reported, as they closely resemble those in Table 6

*Source*: Own elaboration

**Table 9:** Multinomial regression stratified by parity (one child vs. two or more) on fertility intentions and behaviours: relative risk ratios (reference: negative fertility intentions)

Life-term positive intentions	Parity 1		Parity 2 +	
	Model 1	Model 1c	Model 2	Model 2c
*Childcare use* *(regular* + *occasionally)*				
Suboptimal arrangements	0.99	1.15	1.02	0.82
No one to ask	0.86	0.83	1.18	1.42
*Migrant background (Native)*				
Foreign-born	0.73	0.89	0.94	0.72
*Age (Until 24)*				
25–29	0.37***	0.37***	0.63	0.63
30–34	0.90***	0.09***	0.23**	0.23*
35–39	0.03***	0.03***	0.08***	0.08***
40 and more	0.01***	0.01***	0.01***	0.01***
*Childcare use#migratory* *(regular* + *occasionally #Foreign-born)*				
Suboptimal arrangements #Foreign-born		0.38		4.13*
No one to ask #Foreign-born		0.94		0.77
*Area (North)*				
Central	1.17	1.21	1.00	0.98
South	1.13	1.14	0.99	0.98
*Education (Primary)*				
Secondary	1.54*	1.55*	1.74*	1.82*
Tertiary	1.32	1.34	1.83*	1.90*

Short-term intentions				
*Childcare use* *(regular* + *occasionally)*				
Suboptimal arrangements	1.16	1.15	0.97	0.94
No one to ask	1.01	0.77	0.73	0.60*
*Migrant background (Native)*				
Foreign-born	0.59***	0.54***	0.92	0.80
*Age (Until 24)*				
25–29	0.83	0.83	1.23	1.20
30–34	0.52***	0.52**	0.85	0.84
35–39	0.27***	0.27***	0.45	0.44
40 and more	0.76***	0.07***	0.21***	0.20***
*Childcare use#migratory* *(regular* + *occasionally #Foreign-born)*				
Suboptimal arrangements #Foreign-born		1.09		1.34
No one to ask #Foreign-born		1.70		1.67
*Area (North)*				
Central	1.00	0.99	0.74*	0.74*
South	1.16	1.18	0.77*	0.77*
*Education (Primary)*				
Secondary	1.25	1.25	1.11	1.11
Tertiary	1.80***	1.77***	1.46*	1.46*

Pregnant or with child				
*Childcare use* *(regular* + *occasionally)*				
Suboptimal arrangements	0.96	0.92	0.79*	0.78*
No one to ask	1.14	0.58	0.81	0.42**
*Migrant background (Native)*				
Foreign-born	0.54**	0.41***	1.62***	1.22*
*Age (Until 24)*				
25–29	1.15	1.13	0.48**	0.47**
30–34	0.66	0.65	0.19***	0.19***
35–39	0.34***	0.34***	0.07***	0.07***
40 and more	0.05***	0.05***	0.02***	0.02***
*Childcare use#migratory* *(regular* + *occasionally #Foreign-born)*				
Suboptimal arrangements #Foreign-born		1.44		1.03
No one to ask #Foreign-born		3.88**		3.17**
*Area (North)*				
Central	0.85	0.84	0.83	0.83
South	1.05	1.07	1.05	1.07
*Education (Primary)*				
Secondary	1.07	1.04	1.45**	1.45**
Tertiary	2.41***	2.33***	2.74***	2.7***

Data: Birth Sample Survey/Indagine campionaria sulle nascite e le madri 2012

Note: **p* < 0.05; ***p* < 0.01; and ****p* < 0.001

Model 1 is the baseline model without control variables or interactions. Model 1c includes the interaction term between migrant background and childcare use. Model 2 introduces control variables but excludes interaction effects, while Model 2c includes both control variables and the interaction term

*Source*: Own elaboration

## Footnotes

1 The main results of the surveys conducted by ISTAT in 2002, 2005, and 2012 are published in the ebook “Avere figli in Italia negli anni 2000. Approfondimenti dalle indagini campionarie sulle nascite e sulle madri”, Istat, temi, December 2014, http://www.istat.it/it/archivio/147180.

2 As described in the Data and Research Sample section (Sect. 4), mothers who stated that they do not use formal childcare by choice were excluded from the sample.

3 We use the (Rumbaut 2012) classification: 1st-generation migrants (foreign-born, migrated from the age of 18), 1.5-generation (foreign-born, migrated before age 18).
